# Antimicrobial Tear Lipids in the Ocular Surface Defense

**DOI:** 10.3389/fcimb.2022.866900

**Published:** 2022-03-31

**Authors:** Poonam Mudgil

**Affiliations:** School of Medicine, Western Sydney University, Campbelltown, NSW, Australia

**Keywords:** antimicrobial lipids, innate immunity, host defense, ocular, tears

## Abstract

The concept of antimicrobial lipids as effectors of innate host defense is an emerging field. There is limited knowledge on the antimicrobial role of lipids in the ocular environment. Tears act as first line of defense to protect the ocular surface from infections. Antimicrobial effects of tear lipids have been demonstrated using meibomian lipids that are the source of majority of lipids in tears. This article describes the knowledge available on the antimicrobial role of tear lipids at the ocular surface and the antimicrobial potential of various lipid classes present in tears that can contribute to antimicrobial protection of the eye. Like other mucosal secretions, tears contain many proteins and lipids with known antimicrobial effects. The antimicrobial defense of tears is far stronger than can be demonstrated by the effects of individual compounds many of which are present in low concentrations but synergistic and additive interactions between them provide substantial antimicrobial protection to the ocular surface. It is inferred that antimicrobial lipids play important role in innate defense of tears, and cooperative interactions between various antimicrobial lipids and proteins in tears provide a potent host defense mechanism that is effective against a broad spectrum of pathogens and renders self-sterilizing properties to tears for keeping the microbial load low at the ocular surface.

## Introduction

Antimicrobial effects of lipids in the context of ocular environment is scarce in literature. The first report demonstrating the antimicrobial effect of host-derived lipids in the ocular surface defense (Mudgil, 2014) used meibomian lipids that are a complex mixture of lipids secreted from meibomian glands of the eyelids and constitute the majority of lipid component of tears on the surface of the eye. Under physiological conditions similar to the ocular surface, these lipids were antibacterial against several clinical ocular surface pathogens including Gram-positive and Gram-negative bacteria, namely *Staphylococcus aureus*, *Pseudomonas aeruginosa*, and *Serratia marcescens*, and caused extensive cellular damage to bacteria resulting in smaller size, loss of aggregation, abnormal phenotype, cellular distortion, damaged cell wall, and cell lysis (Mudgil, 2014). A possible antimicrobial function for tear lipids was speculated, but never evidenced or substantiated, in some review articles (Tiffany, 1987; Sack et al., 2001; Bron et al., 2004) probably on the basis that meibomian glands are modified sebaceous glands and the similarity in lipid composition of their secretions (meibum Vs sebum) may imply parity in functions, given that antimicrobial role of lipids in sebum is well documented (Drake et al., 2008; Fischer et al., 2014).

Host-derived antimicrobial lipids are increasingly being recognized as part of innate host defense at various mucosal surfaces. The contribution of antimicrobial lipids to host defense is evidenced from many reports on the antimicrobial actions of mucosal secretions as well as effects of individual lipids present in these secretions tested *in vitro* and/or *in vivo* (**Table 1**). Prominent among these is the contribution of antimicrobial lipids in skin. Like tear lipids, skin lipids are complex mixture of non-polar and polar lipids. Skin lipids contain wax monoesters, sterol esters, cholesterol, triglycerides, fatty acids, ceramides, squalene, and sphingosine (Robosky et al., 2008; van Smeden et al., 2014). Fatty acids and sphingosines from skin lipids are potent antibacterial (Bibel et al., 1992; Drake et al., 2008; Fischer et al., 2012; Fischer et al., 2014). Vernix caseosa, the lipid-rich film covering the skin of newborns, contains cholesterol, free fatty acids, ceramides, phospholipids, triglycerides, wax and sterol esters and squalene (Hoeger et al., 2002; Nishijima et al., 2019). Fatty acids in vernix protect neonates from infections (Tollin et al., 2005). The oral mucosal secretions contain cholesterol, fatty acids, triglycerides, wax esters, cholesterol esters and squalene – these lipids are similar to those found in skin secretions but are lower in amounts (Brasser et al., 2011; Wertz, 2021). Sphingosine, sapienic acid and lauric acid are antimicrobial in the oral cavity (Fischer et al., 2013). The secretion of nasal mucosa contains free fatty acids, phospholipids, triglycerides, cholesterol, and cholesterol esters (Do et al., 2008). Cholesterol esters in the nasal fluid contribute to the antimicrobial defense of airways (Do et al., 2008). The lipid profile of human sinus secretion is similar to nasal fluid and contains fatty acids, cholesterol, cholesterol esters, triglycerides (Lee et al., 2010). Cholesteryl esters in sinus fluid play a role in host defense (Lee et al., 2010). Human milk contains triglycerides, phospholipids, cholesterol and fatty acids (Hamosh et al., 1999; Isaacs, 2001). Fatty acids and monoglycerides derived from triglycerides provide protection to infants from infections (Thormar et al., 1987; Hamosh et al., 1999; Isaacs, 2001).

**Table 1:** Antimicrobial lipids in human secretions.

Human secretion	Lipids in the secretion	References (lipid composition)	Antimicrobial lipids in the secretion	References (antimicrobial effect)
Tears	Wax esters, cholesterol esters, mono-, di-, and triglycerides, diesters, free sterols, free fatty acids, hydrocarbons, phospholipids, hydroxyl fatty acids	(Butovich et al., 2007; Chen et al., 2010; Rantamäki et al., 2011; Lam et al., 2014)	Meibomain lipids, oleic acid, cholesterol, cholesterol ester, phospholipid	(daSilva-Antunes, 2013; Mudgil, 2014; Mudgil et al., 2014; daSilva-Antunes et al., 2016)
Skin secretion	Wax monoesters, sterol esters, cholesterol, triglycerides, fatty acids, ceramides, squalene, sphingosine	(Robosky et al., 2008; van Smeden et al., 2014)	Fatty acids, sphingosines	(Bibel et al., 1992; Drake et al., 2008; Fischer et al., 2012; Fischer et al., 2014)
Vernix caseosa	Cholesterol, free fatty acids, ceramides, phospholipids, triglycerides, wax and sterol esters, squalene	(Hoeger et al., 2002; Nishijima et al., 2019)	Fatty acids	(Tollin et al., 2005)
Oral secretion	Cholesterol, fatty acids, triglycerides, wax esters, cholesterol esters, squalene	(Brasser et al., 2011)	Sphingosine, sapienic acid, lauric acid	(Fischer et al., 2013)
Nasal secretion	Free fatty acids, phospholipids, triglycerides, cholesterol, cholesterol esters	(Do et al., 2008)	Cholesterol esters	(Do et al., 2008)
Sinus secretion	Fatty acids, cholesterol, cholesterol esters, triglycerides	(Lee et al., 2010)	Cholesterol esters	(Lee et al., 2010)
Breast milk	Triglycerides, phospholipids, fatty acids	(Hamosh et al., 1999; Isaacs, 2001)	Fatty acids, monoglycerides, hydroxycholesterol, sphingophospholipids	(Isaacs et al., 1986; Thormar et al., 1987; Hamosh et al., 1999; Isaacs, 2001; Sprong et al., 2001; Civra et al., 2019)

## Defense Mechanisms of Tears

The ocular surface has many innate defense mechanisms. The first line of defense is tears that prevents and fights infections by physical and chemical mechanisms. Physical mechanisms include existence of a thin tear film (3-10 μm thick) (Bron et al., 2004) as a barrier to circumvent the direct contact of pathogens with the otherwise vulnerable cornea and the ocular surface. Reflex tearing and washing action of tears further hinder the attachment of pathogens to the ocular surface and wash them away. Tear fluid is continually secreted and drained at an average flow rate of 1.2 µL/min to ensure the continuous cleaning of the ocular surface (Mishima et al., 1966). Reflex stimulation may increase the tear volume by 50-100-fold to quickly get rid of pathogens (Fullard and Tucker, 1991; Tiffany, 2008). Mucins in tears capture, immobilize and remove pathogens in the mucous thread onto the skin (Adams, 1979). Mucins can also serve ‘janitorial’ function by trapping pathogens and removing them from the ocular surface *via* the lacrimal drainage (Gipson et al., 2004).

Chemical mechanisms of defense of tears involve many antimicrobial factors and these are thought to be mainly proteins such as lysozyme, lactoferrin, lipocalin, secretory IgA, complementary factors, secretory phospholipase A_2_, secretory leukocyte protease inhibitor, surfactant protein D, defensins and lacritin, as reviewed comprehensively by McDermott (2013). The knowledge of chemical mechanisms of host defense in tears is somewhat limited to only antimicrobial proteins. Tears contains substantial amount of lipids which are ascribed other important functions but their antimicrobial role is underexplored in comparison to proteins.

## Lipids in Tears

Majority of lipids in tears are derived from meibomian lipids that are secretions of meibomian glands. Meibomian glands are sebaceous holocrine glands located in the upper and lower eyelids. Lipids are produced by the acinar cells of meibomian glands. The acinar cells, after maturation, lyse and release their contents onto the inner margin of eyelids, from where the secreted lipids spread over the aqueous tears forming the outer lipid layer of the tear film (Sirigu et al., 1992; Millar et al., 2010). The composition of meibomian lipids has been studied extensively (Nicolaides et al., 1981; Butovich et al., 2007; Butovich et al., 2009; Chen et al., 2010; Chen, 2021). Tear lipids may contain lipids derived from other sources in addition to those from meibomian lipids. Analysis of meibomian lipids and tear lipids show remarkably similar lipid profiles with the same lipid classes being present in both (Rantamäki et al., 2011; Pucker and Nichols, 2012; Butovich, 2013; Lam et al., 2014), except phospholipids which are more abundant in tear lipids compared to meibum (Dean and Glasgow, 2012; Brown et al., 2013; Lam et al., 2014; Chen et al., 2019). The lipid layer of the tear film is made up of an outer nonpolar layer and an inner polar layer.

The nonpolar lipids in tears include wax esters, cholesterol esters, mono-, di-, and triglycerides, diesters, free sterols, free fatty acids and hydrocarbons. The nonpolar lipids in tears slow down evaporation of the aqueous tears preventing ocular surface inflammation and hyperosmolarity that result from excessive tear evaporation (Bron et al., 2017). Wax esters and cholesterol esters are the main lipid classes making about 80% of total tear lipids. Wax esters contain a long chain fatty acid linked to a long-chain fatty alcohol. The fatty acids and fatty alcohols in wax esters can have various types of branching and unsaturation (Butovich et al., 2007). Different types of fatty acids and alcohols make wax esters quite diverse. Oleic acid is the most prominent fatty acid in wax esters (Butovich et al., 2007; Millar et al., 2010; Butovich, 2013). Cholesterol esters contain cholesterol linked to a long chain fatty acid by an ester bond. Fatty acids in cholesterol esters can be variously branched and unsaturated (Butovich et al., 2007). Wax esters and cholesteryl esters are extremely hydrophobic and have very poor aqueous solubility. Monoglycerides, diglycerides and triglycerides have a glycerol molecule with one, two and three fatty acid chains, respectively. Triglycerides are the most commonly found glycerides (Butovich et al., 2007). Diesters molecules have two ester bonds which can contain either cholesterol, a hydroxy fatty acid and a fatty acid, or two fatty acid and a diol molecule (Chen et al., 2010). Among free sterols in tears, cholesterol is the most commonly found sterol (Harvey et al., 1987; Butovich, 2013). Free cholesterol may be produced by breakdown of cholesterol esters (Butovich et al., 2007; Chen et al., 2010). Among free fatty acids in tears, oleic acid is the most common fatty acids and it may be produced by breakdown of fatty acids-containing lipids (Chen et al., 2010). The hydrocarbon detected in tears is squalene (Butovich, 2008).

The polar lipids in tears include phospholipids and hydroxy fatty acids. Being amphiphilic in nature, polar lipids promote tear film stability by acting as interphase between the nonpolar lipids and the aqueous part of tears allowing the nonpolar lipids to spread over the aqueous tears. Two types of phospholipids are found in tears, glycerophospholipids and sphingophospholipids. These phospholipids are present in appreciable amounts to constitute the amphiphilic polar layer of the tear film (Lam et al., 2014). Glycerophoshpholipids contain a glycerol as a diglyceride with a phosphate group that can be attached to an organic molecule. Sphingophospholipids have a similar structure as glycerophospholipids except they contain sphingosine instead of a diglyceride. The glycerophospholipids reported in tears include phosphatidylcholine, lysophosphatidylcholine, phosphatidylethanolamine, phosphatidylserine, phosphatidic acid, phosphatidylinositol, phosphatidylglycerol (Dean and Glasgow, 2012; Brown et al., 2013; Lam et al., 2014; Chen et al., 2019). The sphingolipids in tears are mainly sphingomyelin and ceramide (Rantamäki et al., 2011; Lam et al., 2014). The hydroxy fatty acids have a fatty acid chain with a hydroxyl group at one end. Tears contain (O-acyl)-ω-hydroxy fatty acids (OAHFA) which are very long-chain ω-hydroxyacids and they form part of the amphiphilic polar layer of the tear film (Butovich et al., 2009; Lam et al., 2014; Chen et al., 2019).

The methodologies for analysis of meibum and tear lipids have been extensively reviewed elsewhere (Butovich, 2009; Pucker and Nichols, 2012; Butovich, 2013). The reliable techniques for identifying lipids have included mass spectrometry-based techniques such as high performance liquid chromatography-mass spectrometry (HPLC-MS), LC-MS with MS fragmentation, and electrospray ionization-mass spectrometry (ESI-MS) (Butovich et al., 2007; Chen et al., 2010; Lam et al., 2014; Chen et al., 2019), while nuclear magnetic resonance is deemed useful for quantification of lipids (Butovich, 2013; Willcox et al., 2017). Tear collection techniques also affect the lipid profiles and indicate that basal tears collection with capillary tubes is a preferred method (Lam et al., 2014; Rentka et al., 2017; Pieczyński et al., 2021). The amounts of various lipid species in tears vary in different studies due to various analytical techniques and collection methods used, and are reviewed elsewhere (Pucker and Nichols, 2012; Butovich, 2013; Pucker and Haworth, 2015; Willcox et al., 2017). Whether the changes in tear lipid composition are associated with susceptibility to infections is not well understood but there are indications that alterations in tear lipids are correlated with the presence of bacteria that produce lipolytic enzyme as reported in chronic blepharitis in human (Dougherty and McCulley, 1986a; Dougherty and McCulley, 1986b) and pink eye infection in cattle (Wood et al., 2018).

## Antimicrobial Potential of Lipid Classes in Tears

**Figure 1:**
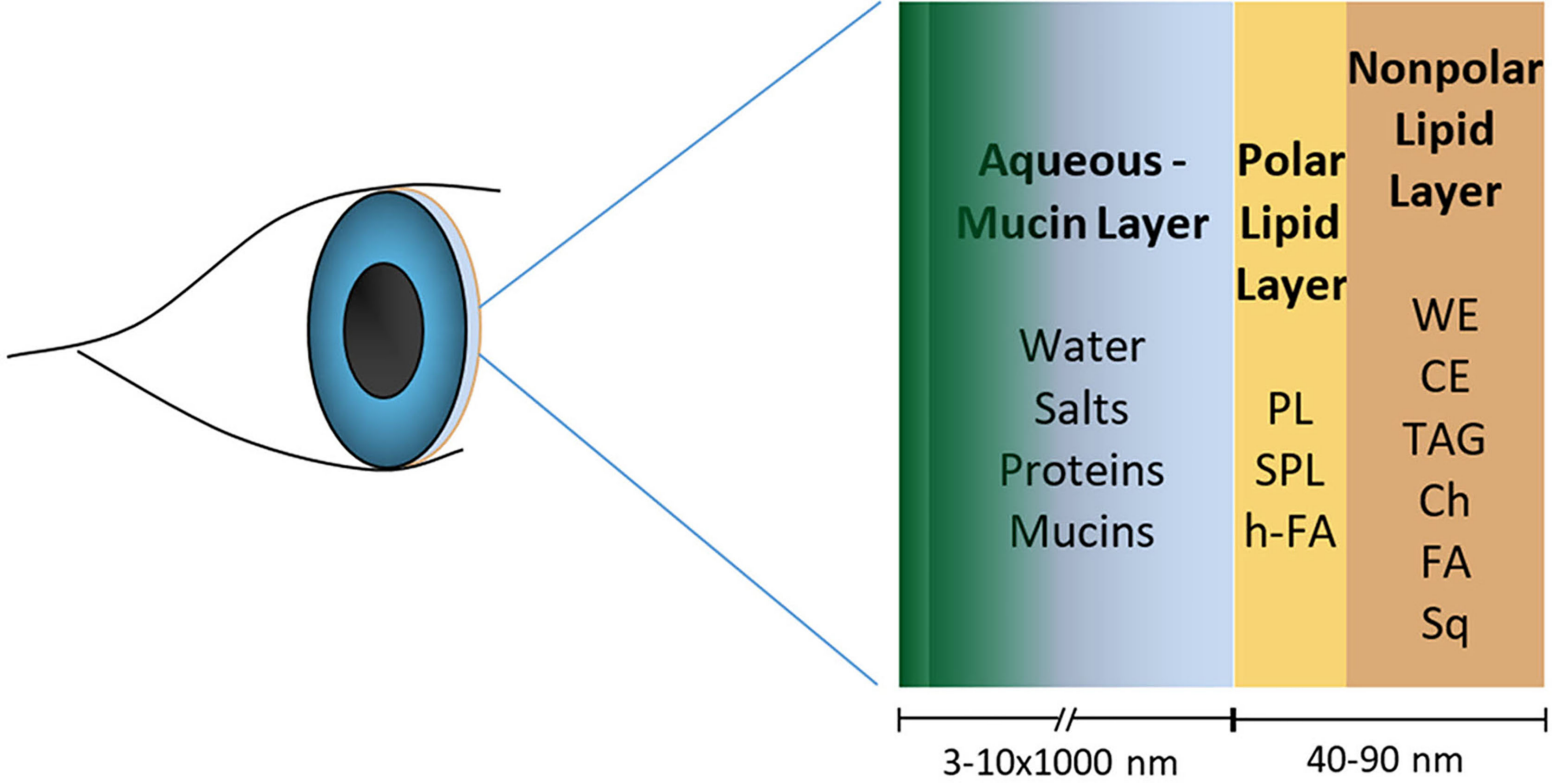
Human tear film model showing the lipid layer with nonpolar and polar lipids that have antimicrobial properties in playing a role in the ocular surface defense. The figure is not to the scale. (WE, wax esters; CE, cholesterol esters; TAG, triglycerides; Ch, free cholesterol; FA, free fatty acids; Sq, squalene; PL, phospholipids; SPL, sphingolipid; h-FA, hydroxy fatty acids).

Various lipid classes present in tears are known to possess antimicrobial properties and have potential to contribute to the antimicrobial defense of tears (**Figure 1** and **Tables 2.1**–**2.3**). Most of the published literature on antimicrobial lipids have tested bacteria, so antibacterial effects are more widely reported than antifungal and antiviral effects. Some of the pathogens mentioned here may not be identified with ocular infections but are included to demonstrate the known antimicrobial potential of various lipid classes as per published reports.

**Table 2.1:** Antimicrobial potential of lipid classes in tears (wax esters, cholesterol esters, mono- and triglycerides, and cholesterol).

Lipid class	Name of lipid	Antimicrobial effect	Target organism	References
Wax esters	Behenyl oleate, behenyl palmitoleate	Antifungal	*Pseudogymnoascus destructans*	(Frank et al., 2018)
Cholesterol esters	Cholesterol oleate	Antibacterial	*Staphylococcus aureus*, *Pseudomonas aeruginosa*	(daSilva-Antunes et al., 2016)
	Cholesteryl linoleate, cholesteryl arachidonate	Antibacterial	*Pseudomonas aeruginosa, Staphylococcus epidermidis*	(Do et al., 2008)
	Cholesteryl linoleate in liposome carrier	Antibacterial	*Staphylococcus epidermidis, Pseudomonas aeruginosa, Enterococcus faecalis*	(Cheung Lam et al., 2016)
Mono-glycerides	Monocaprin	Antibacterial	*Chlamydia trachomatis*, Group A *Streptococcus*, Group B *Streptococcus*, *Staphylococcus aureus*	(Bergsson et al., 1998; Bergsson et al., 2001)
		Antiviral	Herpex simplex virus, repiratory syncytial virus, parainfluenza virus	(Hilmarsson et al., 2005; Hilmarsson et al., 2007)
	Monolaurin	Antibacterial	*Streptococcus pyogenes, Staphylococcus aureus*	(Kabara et al., 1972; Batovska et al., 2009)
		Antiviral	Herpex simplex virus	(Hilmarsson et al., 2005
	Monolinolein		Vesicular stomatitis virus	Thormar et al., 1987)
Triglycerides	Triglycerides	Various	Enveloped viruses, bacteria, protozoa	(Hamosh et al., 1999)
Cholesterol	Cholesterol	Antibacterial	*Staphylococcus aureus*, *Pseudomonas aeruginosa, Serratia marcescens, Streptococcus penumonae*	(Marquart et al., 2007; daSilva-Antunes, 2013)
	25-hydroxy cholesterol	Antiviral	Zika virus	(Tricarico et al., 2019)
	27-hydroxy cholesterol	Antiviral	Rotavirus, rhinovirus	(Civra et al., 2019)

**Table 2.2:** Antimicrobial potential of lipid classes in tears (free fatty acids and hydrocarbons).

Lipid class	Name of lipid	Antimicrobial effect	Target organism	References
Fatty acids	Oleic acid	Antibacterial	MRSA USA 300, *Bacillus megaterium*, *Staphylococcus aureus*, *Pseudomonas aeruginosa, Serratia marcescens, Streptococcus pyogenes*, GAS	(Galbraith et al., 1971; Speert et al., 1979; Zheng et al., 2005; Chen et al., 2011; Mudgil et al., 2014)
		Antiviral	HSV, RSV, VSV, visna virus	(Thormar et al., 1987; Hilmarsson et al., 2005; Hilmarsson et al., 2007)
	Palmitoleic acid	Antibacterial	GAS, GBS, *Staphylococcus aureus, Pneumococcus*, C*orynebacterium* sp.*, Nocardia asteroids, Micrococcus, Streptococcus salivarius, Streptococcus pyogenes*	(Kabara et al., 1972; Bergsson et al., 2001; Wille and Kydonieus, 2003; Zheng et al., 2005; Parsons et al., 2012)
		Antiviral	HSV, RSV	(Hilmarsson et al., 2005; Hilmarsson et al., 2007)
	Linoleic acid	Antibacterial	*Streptococcus faecalis, Bacillus megaterium, Pneumococcus*, GAS, *Corynebacterium* sp.*, Nocardia asteroids, Micrococcus*	(Galbraith et al., 1971; Kabara et al., 1972; Carson & Daneo-Moore, 1980
		Antiviral	VSV, HSV, visna virus	Thormar et al., 1987)
	Linolenic acid	Antibacterial	*Bacillus megaterium*	(Galbraith et al., 1971)
		Antiviral	VSV, HSV, visna virus	(Thormar et al., 1987)
	Sapienic acid	Antibacterial	*Staphylococcus aureus*, *Streptococcus sanguinis, Streptococcus mitis, Fusobacterium nucleatum*	(Fischer et al., 2012; Subramanian et al., 2019)
	Lauric acid	Antibacterial	GAS, GBS, *Staphylococcus aureus, Streptococcus mitis, Streptococcus sanguinis, Corynebacterium striatum, Corynebacterium jeikeium, Pneumococcus*, *Corynebacterium* sp.*, Nocardia asteroids*, MSSA & MRSAs, *Propionibacterium acnes*	(Kabara et al., 1972; Bergsson et al., 2001; Kitahara et al., 2004; Nakatsuji et al., 2009; Fischer et al., 2012)
		Antiviral	HSV, RSV, parainfluenza virus, VSV, visna virus	(Thormar et al., 1987; Hilmarsson et al., 2005; Hilmarsson et al., 2007
	Capric acid	Antibacterial	*Staphylococcus aureus, Chlamydia trachomatis*	Bergsson et al., 1998; Bergsson et al., 2001)
		Antiviral	VSV, HSV, visna virus	(Thormar et al., 1987)
Hydrocarbons	Squalene	Antibacterial	*Sarcina lutea*, *Escherichia coli*	(Biswas and Chakraborty, 2013)
		Antifungal	*Aspergillus*	(Biswas and Chakraborty, 2013)

HSV, Herpes simplex virus; RSV, respiratory syncytial virus; VSV, Vesicular stomatitis virus; GAS, Group A Streptococcus, GBS, Group B Streptococcus; MSSA, Methicillin-susceptible Staphylococcus aureus (MSSA); MRSA, Methicillin-resistant Staphylococcus aureus.

**Table 2.3:** Antimicrobial potential of lipid classes in tears (phospholipids and hydroxyl fatty acids).

Lipid class	Name of lipid	Antimicrobial effect	Target organism	References
Phospholipids	Phosphatidyl choline	Antibacterial	*Staphylococcus aureus*, *Pseudomonas aeruginosa*	(daSilva-Antunes et al., 2016)
	Lyso phospholipid	Antibacterial	*Pseudomonas aeruginosa*	(Krogfelt et al., 2000; Laux et al., 2002)
	Phosphatidyl glycerol	Antiviral	Respiratory syncytial virus, influenza A virus	(Numata et al., 2010; Numata et al., 2012)
	Phosphatidyl inositol	Antiviral	Respiratory syncytial virus	(Numata et al., 2015)
	Oxidized phospholipids	Antiviral	Vesicular stomatitis virus	(Ernandes and Kagan, 2021)
	Sphingolipids	Antibacterial	*Escherichia coli, Salmonella enteritidis, Campylobacter jejuni, Listeria monocytogenes*	(Sprong et al., 2001)
	Sphingosine	Antibacterial	*Streptococcus pyogenes, Micrococcus luteus, Propionibacterium acnes, Brevibacterium epidermidis*, *Candida albicans, Escherichia coli, Streptococcus mitis, Staphylococcus aureus, Streptococcus sanguinis, Corynebacterium bovis, Corynebacterium striatum, Corynebacterium jeikeium, Fusobacterium nucleatum, Pseudomonas aeruginosa, Acinetobacter baumannii, Moraxella catarrhalis*	(Bibel et al., 1992; Fischer et al., 2012; Pewzner-Jung et al., 2014; Tavakoli Tabazavareh et al., 2016)
	Ceramide	Antibacterial	*Neisseria meningitides*, *Neisseria gonorrhoea*	(Becam et al., 2017)
Hydroxy fatty acids	Hydroxy fatty acids	Antibacterial	*Bacillus subtilis*, *Listeria monocytogenes, Staphylococcus aureus*, *Pseudomonas aeruginosa*	(Shin et al., 2004; Desbois and Lawlor, 2013)
	Hydroxy polyunsaturated fatty acids	Antiviral	Influenza virus	(de Toledo-Piza et al., 2018)

### Wax Esters

Wax esters protect tears from evaporative stress and help in preventing drying of the ocular surface. Being present in the outermost layer of the tear film, they serve as a mechanical barrier to microbial invasion. Wax esters are characteristically present in tear lipids produced by meibomian glands, and in skin lipids produced by sebaceous glands (Robosky et al., 2008; Smith and Thiboutot, 2008). Their presence on the outer surface of the body, such as skin, has a barrier function that provides protection from the outside environment and desiccation. Wax esters such as behenyl oleate and behenyl palmitoleate have been shown to have antifungal properties (Frank et al., 2018). The antibacterial properties of wax esters are not widely documented in literature, possibly due the fact that their extreme hydrophobicity makes it difficult to conduct conventional *in vitro* antimicrobial assays where bacterial pathogens need to be grown and tested in aqueous media.

### Cholesterol Esters

Cholesterol ester inhibit growth of ocular pathogenic bacteria *S. aureus* and *P. aeruginosa* but do not kill these bacteria as revealed in an *in vitro* study using physiological conditions of tears (daSilva-Antunes et al., 2016). Cholesterol esters contribute to the inherent antibacterial activity of human nasal mucosa and Do et al. (2008) have demonstrated that cholesteryl linoleate and cholesteryl arachidonate exhibit direct antibacterial activity against *P. aeruginosa* and *S. epidermidis in vitro*, with cholesteryl linoleate being a more potent antibacterial lipid with a broader spectrum. Elevated levels of cholesteryl esters observed in sinus secretions of chronic rhinosinusitis patients (Lee et al, 2010) and bronchoalveolar lavage fluid of cystic fibrosis patients (Ma et al., 2015) indicate contribution of cholesterol esters to the innate host defense of the respiratory tract. In addition, cholesteryl linoleate in a liposome carrier has been shown to exhibit antibacterial activity against *S. epidermidis* and *P. aeruginosa* and lowering the minimum inhibitory concentration of vancomycin for vancomycin resistant *Enterococcus faecalis* (Cheung Lam et al., 2016).

### Mono- and Triglycerides

Glycerides have antibacterial and antiviral properties. Monoglycerides containing linoleic acid show antiviral effects against enveloped vesicular stomatitis virus and cause reduction in virus titre in the antiviral activity assays *in vitro* (Thormar et al., 1987). Monocaprin, a monoglyceride containing capric acid, has been shown to be effective in killing three Gram-positive bacteria: Group A *Streptococcus* (GAS), Group B *Streptococcus* (GBS) and *S. aureus*. The study of the mode of action of monocaprin against GBS showed that the mechanism of killing was disruption of cell membrane because electron micrographs of bacteria treated with the lipid showed disintegrated cell membrane with cell wall left intact. It was suggested that the highly lethal effect of this monoglyceride could be utilized for treating infections caused by GBS (Bergsson et al., 2001). In an earlier work from the same group, monocaprin was shown to inactivate *Chlamydia trachomatis* by disrupting the membrane of the elementary bodies of the bacteria suggesting use of this lipid as a microbicidal agent (Bergsson et al., 1998). Monocaprin are also virucidal against herpex simplex virus, respiratory syncytial virus and parainfluenza virus (Hilmarsson et al., 2005; Hilmarsson et al., 2007). Another monoglyceride, monolaurin, shows a high antibacterial potency against *S. aureus* that is even greater than the antimicrobial effect of the fatty acid, lauric acid, derived from it (Kabara et al., 1972). Synergistic activities of monoglycerides have also been observed against Gram-positive bacteria, *Streptococcus pyogenes* and *S. aureus* (Batovska et al., 2009). Antimicrobial properties of monoglycerides and their fatty acids against various bacterial species suggest their possible therapeutic applications as alternative to antibiotics for combating infections (Churchward et al., 2018; Yoon et al., 2018).

Triglycerides are abundantly present in the human milk fat globules. Triglycerides upon hydrolysis by gastric lipases in the stomach of newborns produce free fatty acids and monoglycerides that can lyse the enveloped viruses, bacteria, and protozoa (Hamosh et al., 1999). Monoglycerides act additively with fatty acids and their combined concentration determines the antimicrobial lipid activity of human milk in which microbial inactivation happens by membrane destabilization (Isaacs, 2001). Similarly, triglycerides in skin lipids serve as source of fatty acids that act as potent antimicrobials at the skin surface (Drake et al., 2008; Fischer et al., 2014)

### Cholesterol

Cholesterol being the main sterol in tear lipids was tested in an *in vitro* study and it inhibited the growth of clinical strains of eye pathogens, *S. aureus*, *P. aeruginosa* and *S. marcescens*, at low concentrations but it showed a little or no inhibitory effect at high concentrations (daSilva-Antunes, 2013). In this study, cells treated with cholesterol showed abnormal phenotype and loss of cellular content in scanning electron micrographs (daSilva-Antunes, 2013). In a previous study, Marquart et al. (2007) reported 1% cholesterol to be bactericidal against *Streptococcus pneumonia in vitro* and lower concentration of cholesterol being partially inhibitory in a concentration dependent manner. They proposed that topical application of cholesterol might be useful for the treatment of *S. penumonae* keratitis because cholesterol can inhibit pneumolysin and kill bacteria. Another study shows that targeted regulation of membrane cholesterol content is used as a host defense strategy to evade bacterial toxins that damage the animal cells by pore formation in the cell membrane (Zhou et al., 2020).

Oxysterols, the oxidation derivatives of cholesterol, have broad antiviral activity against enveloped and non-enveloped human viral pathogens (Lembo et al., 2016). Two oxysterols, namely 25-hydroxycholesterol and 27-hydroxycholesterol, possess broad antiviral activity and are involved in innate antiviral defense. 25-hydroxycholesterol has antiviral activity against Zika virus (Tricarico et al., 2019). 27-hydroxycholesterol present in colostrum is effective against paediatric viral pathogens, rotavirus and rhinovirus, suggesting that breastfeeding helps in transfer of protective factors to infants in the initial days of lactation (Civra et al., 2019). Cholesterol and its oxysterols also modulate the hepatic innate immune response against Hepatitis C virus (HCV) infection. It is proposed that cholesterol modifications can be used for adjuvant therapy and clinical management of patients with HCV infection (González-Aldaco et al., 2018).

### Fatty Acids

Tears contain about 2% of free fatty acids and out of these oleic acid is the main fatty acid reported, although the amounts reported in literature vary a lot and are dependent on the analytical techniques used (Chen et al., 2010). In our previous study, oleic acid inhibited growth of clinical strains of eye pathogens, *S. aureus*, *P. aeruginosa* and *S. marcescens* in a concentration dependent manner with 1% concentration showing complete growth inhibition (Mudgil et al., 2014). Cells treated with oleic acid showed cellular distortions and cell lysis in scanning electron micrographs. Given its antimicrobial activity, oleic acid can be used to develop lipid-based treatment for eye infections helping in reducing antibiotics usage. Antibacterial activity associated with tear lipids identified in these bacteria may be relevant to other Gram-positive and Gram-negative bacteria with applications in treating a range of bacterial ocular infections. The mechanism by which oleic acid or other unsaturated fatty acids such as linoleic acid or palmitoleic acid may exhibit antibacterial action is by inhibition of bacterial fatty acid synthesis. These unsaturated fatty acids inhibit bacterial enoyl-acyl enoyl-acyl carrier protein reductase (FabI) which is an essential component of bacterial fatty acid synthesis (Zheng et al., 2005). FabI is responsible for catalysis of the final and rate-limiting step of the fatty acid chain elongation in bacteria. Inhibition of FabI by oleic acid and palmitoleic acid is noted against *S. aureus* and *S. pyogenes* but these lipids are not effective against *Escherichia coli* or *P. aeruginosa* (Zheng et al., 2005). Oleic acid and linoleic acid, also induce cell and protoplast lysis of *Streptococcus faecalis* by acting as membrane destabiliser (Carson and Daneo-Moore, 1980).

Oleic acid is also found in skin lipids and is known to be antibacterial against skin pathogens including methicillin-resistant *S. aureus* (MRSA) and group A streptococci (Speert et al., 1979; Chen et al., 2011). It kills *S. aureus* bacteria by breaking down the cell walls and is effective against many *S. aureus* strains including the multi-antibiotic resistant community associated MRSA USA 300 (Chen et al., 2011). Palmitoleic acid is another antimicrobial fatty acid in mammalian skin that protects against *S. aureus* and Gram-positive bacterial infections (Wille and Kydonieus, 2003; Parsons et al., 2012). It permeabilizes the cell membrane causing leakage of solutes and low-molecular-weight proteins into the medium (Parsons et al., 2012). Free fatty acids provide defense against *S. aureus* in healthy skin and their deficiency increases vulnerability of atopic dermatitis patients to colonization by *S. aureus* (Takigawa et al., 2005). They also create unfavorable growth conditions for bacteria by making the skin surface acidic (Fluhr et al., 2001). Sapienic acid is the major antimicrobial fatty acid uniquely present in human skin that arrests growth of *S. aureus* by countering the bacterial defense mechanisms (Subramanian et al., 2019). Sapienic acid has been shown to be antibacterial against *Streptococcus sanguinis, Streptococcus mitis, and Fusobacterium nucleatum* but not against *E. coli, S. aureus, S. marcescens*, and *P. aeruginosa* (Fischer et al., 2012). Lauric acid found in skin is a very potent antimicrobial and shows antibacterial activity against Gram-positive bacteria including *S. aureus, S. mitis, S. sanguinis, Corynebacterium striatum*, and *Corynebacterium jeikeium*, but it is not active against Gram-negative bacteria *E. coli, S. marcescens, or P. aeruginosa*, although it is active against *F. nucleatum* (Fischer et al., 2012). Lauric acid was found as the most potent fatty acid among a number of saturated fatty acids investigated against methicillin sensitive and resistant *S. aureus* (Kitahara et al., 2004). The strong antimicrobial effects of lauric acid against *Propionibacterium acnes in vitro* and *in vivo* show that it can be used as an alternative treatment for antibiotic therapy of acne vulgaris (Nakatsuji et al., 2009). A number of fatty acids including lauric acid and capric acid are antibacterial against GAS, GBS, *S. aureus*, and *C. trachomatis* (Bergsson et al., 1998; Bergsson et al., 2001), Fatty acids are also virucidal against herpes simplex virus and their activity increases even more in the acidic environment (Hilmarsson et al., 2005).

Host-derived fatty acids found in human milk play an important role in providing innate defense to newborns and infants. Antiviral activity in the human milk appears after storage at 4°C for 2 days. It reduces viral titre by as much as 10,000-fold and is due to antiviral fatty acids in the milk (Isaacs et al., 1986). The medium-chain saturated and long-chain unsaturated fatty acids in human milk are active against enveloped viruses vesicular stomatitis virus, herpes simplex virus, and visna virus *in vitro*. The antiviral activity results in the leakage of the viral envelope, and at higher concentration of fatty acids there is a complete disintegration of the envelope and the viral particles (Thormar et al., 1987). Fatty acids may exert a detergent-like effect on lipid-coated microbes. They can incorporate into the lipid membrane causing instability, which in turn results in the rupture of the membrane and death of the organism (Isaacs et al., 1986; Thormar et al., 1987).

Fatty acids typically have broad-spectrum antimicrobial effects. Unsaturated fatty acids generally have more antimicrobial effects than saturated fatty acids (Kabara et al., 1972; Zheng et al., 2005). Investigating antimicrobial effects of a variety of fatty acids, Kabara (1984) concluded that saturated fatty acids have highest activity when the chain length is C12 (lauric acid), monounsaturated fatty acids have highest activity in parmitoleic acid and the most active polyunsaturated fatty acid is linoleic acid. Another study with different Gram-positive bacteria indicated that unsaturated fatty acids having C18 chains such as oleic acid, linoleic acid and linolenic acid have potent antimicrobial activities (Galbraith et al., 1971). It is known that medium- and long-chain unsaturated fatty acids generally have more antimicrobial effects against Gram-positive bacteria in comparison to Gram-negative bacteria (Galbraith et al., 1971; Chen et al., 2011). The outer membrane of Gram-negative bacteria protects them from the destructive action of fatty acids (Speert et al., 1979). Fatty acids packaged in liposomes can be promising lipophilic antimicrobial agents. Palmitic acid and steric acid in liposome preparations have shown antibacterial activity against multidrug resistant *S. epidermidis* and vancomycin resistant *E. faecalis* (Cheung Lam et al., 2016). Lauric acid incorporated in a liposome kills *P. acnes*. The liposome fuses with the membrane of bacteria and releases the fatty acid directly on the bacterial membranes to kill the bacteria efficiently (Yang et al., 2009).

A number of mechanisms have been proposed for the antimicrobial action of fatty acids. They mainly target the bacterial cell membrane and affect cellular protection and functions (Desbois and Smith, 2010; Yoon et al., 2018). The proposed mechanisms include (1) exhibiting deleterious detergent effects on the cell membrane causing pore formation, leakage, and cell lysis, (2) interfering with the cellular energy production by disrupting the electron transport chain and uncoupling oxidative phosphorylation, (3) inhibiting membrane enzymes activity and nutrient uptake, and (4) formation of hydroperoxides causing oxidative stress. Fatty acids can insert into the bacterial cell membrane and increase its permeability. This membrane-lytic action causes destabilization, pore formation, leakage of contents and cell lysis (Greenway and Dyke, 1979; Speert et al., 1979; Carson and Daneo-Moore, 1980; Chamberlain et al., 1991). The electron transport chain in the cell membrane of bacteria is the site for energy production. Medium- and long-chain saturated and unsaturated fatty acids can disrupt the electron transport chain by binding to the electron carriers resulting in reduced energy production (Galbraith and Miller, 1973; Kenny et al., 2009). Fatty acids can further reduce energy production through uncoupling of oxidative phosphorylation by decreasing the membrane potential and proton gradient, or by directly binding with the ATP synthase (Sheu and Freese, 1972; Galbraith and Miller, 1973). Fatty acids can directly inhibit membrane enzymes and target membrane-associated proteins in bacteria, for example, inhibiting glucosyl transferase affecting glucan production (Won et al., 2007), and inhibiting enoyl-acyl enoyl-acyl carrier protein reductase (FabI) affecting fatty acid synthesis (Zheng et al., 2005). These effects are generally greater for unsaturated fatty acids than saturated fatty acids (Zheng et al., 2005). Fatty acid can starve bacteria by inhibiting their ability to uptake nutrients such as amino acids (Galbraith and Miller, 1973). Formation of hydroperoxides causing oxidative stress is another mechanism suggested for the bactericidal effects of polyunsaturated fatty acids (Knapp and Melly, 1986).

### Squalene

Squalene shows antimicrobial activities against bacteria, *Sarcina lutea* and *E. coli*, and fungi causing aspergillosis (Biswas and Chakraborty, 2013). Squalene also has anti-oxidative properties and used as adjuvant in vaccines and cosmetics (Fox, 2009). Squalene in microemulsions has been shown to be antimicrobial against MRSA (Fang et al., 2019), and effective in the treatment of COVID-19 patients (Ebrahimi et al., 2022).

### Phospholipids

The *in vitro* studies with the ocular pathogenic bacteria *S. aureus* and *P. aeruginosa* show that phosphatidylcholine (PC) inhibits bacterial growth slightly but time kill assays indicate that it does not kill these bacteria (daSilva-Antunes et al., 2016). Lysophosphatidic acid (LPA), a polar lipid involved in cell proliferation and wound healing, has a protective role in the activation of innate immune response and it enhances antimycobacterial activity both *in vitro* and *ex vivo* (Garg et al., 2006). LPA is present in solution form bound to albumin in many extracellular fluids including aqueous humor in the eye and is released *in vitro*. It is termed as a ‘bioactive’ phospholipid whose receptors and metabolic enzymes can be promising pharmacological targets in finding relevance of bioactivity of LPA *in vivo* (Saulnier-Blache, 2004).

Phospholipids can themselves be inhibitory to bacterial growth or enhance activity of antibiotics against antibiotic-resistant bacteria by increasing permeability of outer membrane to antibiotics through their ability to chelate divalent cations. *P. aeruginosa* is an opportunistic pathogen that causes chronic lung infection in patients with cystic fibrosis. Biofilm formation by this bacteria enhances development of cystic fibrosis. Monopalmitoylphosphatidic acid (MPPA), a host-derived lysophospholipid that accumulates in inflammation, has been shown to slow the growth of antibiotic-resistant strains of *P. aeruginosa* isolated from sputum of cystic fibrosis patients (Krogfelt et al., 2000). It hinders pathogenesis of *P. aeruginosa* PAO1 by inhibiting bacterial virulence factors such as extracellular accumulation of alginate, elastase, LasA protease, and siderophore pyoverdin, as well as biofilm formation (Laux et al., 2002). The inhibitory effect of MPPA is partly attributed to its ability to bind divalent cations and to physically disrupt the bacterial membrane structure. Phospholipids can also enhance the activity of β-lactam antibiotics against *P. aeruginosa* strains (Krogfelt et al., 2000). MPPA enhances activity of ampicillin *in vitro* against *P. aeruginosa* PAO1 by chelating divalent cations, and enhances the activity of pireracillin and ceftazidime against *P. aeruginosa* strains isolated from cystic fibrosis patients (Krogfelt et al., 2000). Liposomes containing bioactive lipids such as phosphatidic acid and phosphatidylinositol stimulate pulmonary cells to kill drug-resistant bacterial pathogens by augmenting immune response showing that liposomes delivered bioactive lipids enhance antimicrobial response and can be used as an additional host-directed strategy for the control of chronic drug-resistant infections (Poerio et al., 2017).

The anionic surfactant lipids of the lung, phosphatidylglycerol (PG) and phosphatidylinositol (PI), exert potent antiviral activities *in vitro* and *in vivo* against respiratory viruses including respiratory syncytial virus and influenza A virus (Numata et al., 2010; Numata et al., 2012; Numata et al., 2015). PG and PI thus paly complementary role in innate immune antiviral defense in the lung. Surfactant preparations containing PG can be used for treating respiratory viral infections and potentially for improving lung function in COVID-19 patients (Bollag and Gonzales, 2020; Ji et al., 2021).

Oxidized phospholipids that are naturally released from dead cells block the replication of RNA viruses during the early stage of viral infection in human epithelial cells (Ernandes and Kagan, 2021). Slowing viral growth prior to infection by oxidized phospholipids allows time for other immune mechanisms to take over, hence, helping in innate host defense against RNA viral infections. Endogenously produced oxidized phospholipids inhibit inflammation and provide protection from lethal endotoxin shock in severe Gram-negative bacterial infections showing that oxidized phospholipids that have ability to inhibit endotoxins can be used for developing drugs for sepsis (Bochkov et al., 2002).

### Sphingolipids

Sphingomyelin, the phospholipid or more specifically a sphingophospholipid, present in human milk provides protection to neonates from bacterial infections (Silva et al., 2021). Bactericidal activity of sphingolipids of milk lipids against pathogenic strains of *E. coli, Salmonella enteritidis, Campylobacter jejuni, Listeria monocytogenes* has been shown *in vitro* (Sprong et al., 2001).

Sphingosines, the base in the sphingophospholipid molecules, are potent broad acting antimicrobial present on the skin and constitute the innate immune defense of skin (Drake et al., 2008; Fischer et al., 2014). It is proposed that exogenous application of these lipids to skin can be a therapeutic option for people at risk of infection (Fischer et al., 2014). Reduced levels of sphinogosine is associated with vulnerability of atopic dermatitis patients to colonization by *S. aureus* (Arikawa et al., 2002). The antibacterial activity of sphingosine has been shown *in vitro* against many Gram-negative bacteria and Gram-positive bacteria including *E. coli, S. mitis, S. aureus, S. sanguinis, Corynebacterium bovis, C. striatum, C. jeikeium*, and *F. nucleatum* but not against *S. marcescens* and *P. aeruginosa* (Fischer et al., 2012). Sphingosines are also antibacterial against *S. pyogenes, Micrococcus luteus, P. acnes, Brevibacterium epidermidis*, and *Candida albicans in vitro* (Bibel et al., 1992). The bactericidal activity of sphingosine against *S. aureus* has been shown *in vitro*, and the *in vivo* infections in mice indicate that lack of sphingosine causes susceptibility to lung infection by *Staphylococcus aureus* in cystic fibrosis (Tavakoli Tabazavareh et al., 2016). Sphingosine is directly involved in pathogenic defense and provides protection from *P. aeruginosa* infections (Baker et al., 2018). *In vitro* studies indicate that sphingosines are antibacterial against *P. aeruginosa, Acinetobacter baumannii*, and *Moraxella catarrhalis*, and *in vivo* studies in mice indicate that sphingosine can prevent lung infection by *P. aeruginosa* in cystic fibrosis patients (Pewzner-Jung et al., 2014).

Ceramides are derived from sphingomyelins and they have been shown to regulate mammalian defense against *P. aeruginosa* and *S. aureus* pathogens that are commonly found in pneumonia (Baker et al., 2018). Ceramides also have potent bactericidal activity against pathogenic Neisseriae. Antibacterial activity of ceramides has been observed against *Neisseria meningitidis* and *Neisseria gonorrhoeae in vitro* with kinetic assays showing killing of *N. meningitidis* within 2 h (Becam et al., 2017).

### Hydroxy Fatty Acids

OAHFAs found in tears are also found in equine amniotic fluid and semen (Wood, 2020), and vernix caseosa in newborns (Kalužíková et al., 2017). OAHFAs act as surfactants in these fluids. While antimicrobial action of OAHFAs is not well reported, antimicrobial properties of other hydroxyl fatty acids are known. Hydroxy polyunsaturated fatty acids exert antiviral activity against influenza virus by interfering with the binding of virus to host cell receptors and reducing viral titres (de Toledo-Piza et al., 2018). Hydroxy fatty acids also show antibacterial activities against Gram-positive bacteria, *Bacillus subtilis*, *L. monocytogenes, S. aureus*, and Gram-negative bacteria, *P. aeruginosa* (Shin et al., 2004; Desbois and Lawlor, 2013).

## Mode of Antimicrobial Action of Tear Lipids

There is lack of understanding on the mode of antimicrobial action of tear lipids. Additionally, the mechanism of action is difficult to predict due the mixed nature of tear lipids. However, the current knowledge on the action of various lipids classes such as detergent effects of fatty acids (Desbois and Smith, 2010) and virulent and direct killing effects of cholesterol and phospholipids (Laux et al., 2002; Marquart et al., 2007), with the known surfactant properties of tear lipids (Mudgil and Millar, 2011) indicate that membrane destabilization by surfactant/detergent activity may be a likely mechanism of antimicrobial action of tear lipids (Mudgil, 2014). It is also probable that various lipid classes in tear lipids exhibit multiple modes of action and together they add to the overall antimicrobial effects of tear lipids.

## Host Defense as a Cooperative Action of Antimicrobial Lipids and Antimicrobial Proteins

Though tears are known to contain many antimicrobial proteins (McDermott, 2013), none of them is a potent antimicrobial on its own. They are effective in combination with each other and with antimicrobial lipids, and the host defense relies on the cooperative interactions between antimicrobial lipids and proteins (Mudgil, 2014). Many antimicrobials with multiple mode of actions help tears evade a broad array of pathogens and cooperative interactions between these antimicrobials makes them effective at lower concentrations providing self-sterilizing properties to tears with characteristically low microbial load. Cooperative interactions of antimicrobial lipids and proteins contributing to innate host defense is also applicable to other body secretions such as breast milk and secretions of skin, oral, nasal and lung mucosa.

Antimicrobial activities of human milk results from protective factors acting individually, additively and synergistically (Isaacs, 2005). Triglycerides in human milk release antimicrobial free fatty acids and monoglycerides which act additively for the overall lipid-dependent antimicrobial activity (Isaacs, 2001). Antimicrobial milk lipids further act synergistically with antimicrobial peptides to decrease the concentrations of individual compounds required for protection and reduce the time needed for inactivation of pathogens showing that synergies of lipids and proteins provide powerful protection from simple compounds at lower concentrations (Isaacs, 2005; Newburg, 2005). The overall antimicrobial protection from human milk is, therefore, far greater than can be demonstrated by effects of antimicrobial factors individually (Isaacs, 2005).

Antimicrobial synergy between lipids and proteins is a part of innate immunity of human skin (Brogden et al., 2012). Antimicrobial synergy occurs between free fatty acids of sebum and histone H4 of sebocytes against *S. aureus* (Lee et al., 2009). Synergistic interactions of sphingosine with cathelicidin and LL37 against a range of Gram-positive bacteria, Gram-negative bacteria, and yeast have been noted (Robertson et al., 2006). Free fatty acids in skin lipids not only provide direct antibacterial activities but they enhance the antimicrobial defense of skin by inducing the expression of antimicrobial peptides in sebocytes. Incubation of sebocytes with lauric acid, palmitic acid, or oleic acid profoundly enhances expression of human β-defensin of sebocytes which shows activity against *P. acnes* suggesting that free fatty acids in skin lipids upregulate the expression of β-defensin in sebocytes (Nakatsuji et al., 2010). Similarly, short chain fatty acids enhance expression of antimicrobial protein, human cathelicidin LL-37 in colonocytes and play a role in mucosal immune defense (Schauber et al., 2003).

Vernix caseosa, the creamy substance covering skin of newborns, is another example of innate host defense that is based on the cooperative interactions between antimicrobial lipids and proteins (Tollin et al., 2005). Free fatty acids in vernix exhibit antimicrobial activities and vernix lipids further enhance the activity of antimicrobial peptides indicating strong host defense resulting from interactions between antimicrobial lipids and proteins that provides protection to foetus and newborn against infections.

The secretion of oral mucosa contains many antimicrobial salivary proteins (lysozyme, lactoferrin, lactoperoxidase), antimicrobial salivary peptides (defensins, cathelicidins, histatins), and antimicrobial salivary lipids (fatty acids derived from salivary triglycerides and long-chain bases from oral epithelial sphingolipids), and together these antimicrobial factors determine the microbial composition of the oral cavity (Wertz and de Szalay, 2020). The secretion of nasal mucosa harbours many antimicrobial proteins and lipids. Lipids in the nasal fluid show synergistic effects with the antimicrobial peptide, human neutrophil peptide HNP2, against *P. aeruginosa* (Do et al., 2008). The elevated expression of antimicrobial factors in the sinus tissue of chronic rhinosinusitis patients may represent a concerted intrinsic defense response in which antimicrobial lipids and antimicrobial proteins act synergistically to combat offending pathogens (Lee et al., 2014). Lysozyme is a prominent antimicrobial protein in lung mucosa. Patients with cystic fibrosis get frequent lung infections with *P. aeruginosa* and supplementation of a non-esterified fatty acid, docosahexaenoic acid, improves clinical condition in these patients. Synergistic activity of human lysozyme and docosahexaenoic acid has been observed against *P. aeruginosa* in which the fatty acid facilitates incorporation of lysozyme into the bacterial membrane allowing influx of more fatty acid that leads to the bacterial cell death (Martinez et al., 2009).

The contribution of antimicrobial lipids to overall intrinsic host defense is further emphasised by association of decreased lipid levels with lowered host defense. The antimicrobial activity of nasal fluid decreases upon depleting the lipids and is restored after re-supplementing the lipids (Do et al., 2008). Decrease in levels of fatty acids and sphingosine in atopic dermatitis patients is associated with their vulnerability to colonization by *S. aureus* (Arikawa et al., 2002; Takigawa et al., 2005). Reduced levels of sphingosine in tracheal and bronchial epithelial cells are associated with susceptibility to lung infection by *P. aeruginosa* in cystic fibrosis patients (Pewzner-Jung et al., 2014). Children feeding on low fat milk are more susceptible to gastrointestinal infection in comparison with those feeding on whole milk (Koopman et al., 1984). Addition of medium chain monoglycerides to human milk and infant formulas can provide increased protection to infants from infections by respiratory syncytial virus, herpes simplex virus type 1, *Haemophilus influenzae*, and GBS (Isaacs et al., 1995).

In addition to the cooperative interactions between lipids and proteins, lipidation increases the antimicrobial activity of peptides involved in innate defense. Potency of antimicrobial peptides can be enhanced by conjugation with fatty acids and sometimes inactive peptides can be rendered active (Li et al., 2013). Fatty acid conjugation enhances the interaction of peptide with the microbial membranes that helps in exerting enhanced antimicrobial effect. Lipidated analogs of peptides generated by conjugation of human peptide with fatty acids provide antimicrobial activity against ESKAPE bacteria and biofilms of *S. aureus* (Kamysz et al., 2020). An engineered short lipopeptide generated by conjugating human cathelicidin LL-37, an innate immune antimicrobial peptide, with fatty acids shows robust antimicrobial activity against Gram-positive and Gram-negative bacteria *in vitro* and reduces bacterial burden of MRSA in mice *in vivo* (Lakshmaiah Narayana et al., 2021). It targets bacterial membranes and makes it helical so that bacteria find it difficult to develop resistance. The designer lipopeptie also has antibiofilm and immune modulation activities as it prevents biofilm formation in a catheter-associated mouse model and recruits cytokines to clear infection near catheters. Fatty acylation of an inactive human β-defensin generates a highly active peptide with potent antimicrobial activity against bacteria and *C. albicans* (Sharma et al., 2015). Fatty acylation increases hydrophobicity and potency of the peptide by allowing greater interaction of the peptide chain with the microbial cell surface that causes membrane permeabilization.

## Conclusion

The knowledge on antimicrobial lipids in tears at the ocular surface is sparse. Meibomian lipids that form majority of tear lipids have been shown to be antimicrobial and various lipid classes present in tears are known to possess antimicrobial properties indicating the importance of antimicrobial lipids in innate immunity of tears in protecting the ocular surface from infections. Akin to other body secretions, the overall defense mechanism of tears is based on the synergistic interactions between antimicrobial lipids and antimicrobial proteins that is effective against a broad spectrum of pathogens and renders self-sterilizing properties to tears for keeping the microbial load low at the ocular surface.

## Author Contributions

The author confirms being the sole contributor of this work and has conceptualized, investigated and analyzed the current research, wrote, reviewed, and edited this article, and approved it for publication.

## Conflict of Interest

The author declares that the research was conducted in the absence of any commercial or financial relationships that could be construed as a potential conflict of interest.

## Publisher’s Note

All claims expressed in this article are solely those of the authors and do not necessarily represent those of their affiliated organizations, or those of the publisher, the editors and the reviewers. Any product that may be evaluated in this article, or claim that may be made by its manufacturer, is not guaranteed or endorsed by the publisher.

## References

[B1] Adams A. S. (1979). The Morphology of Human Conjunctival Mucin. Arch. Ophthalmol. 97, 730–734. doi: 10.1001/archopht.1979.01020010382023 426693

[B2] Arikawa J. Ishibashi M. Kawashima M. Takagi Y. Ichikawa Y. Imokawa G. (2002). Decreased Levels of Sphingosine, a Natural Antimicrobial Agent, may be Associated With Vulnerability of the Stratum Corneum From Patients With Atopic Dermatitis to Colonization by *Staphylococcus Aureus* . J. Invest. Dermatol. 119, 433–439. doi: 10.1046/j.1523-1747.2002.01846.x 12190867

[B3] Baker J. E. Boudreau R. M. Seitz A. P. Caldwell C. C. Gulbins E. Edwards M. J. (2018). Sphingolipids and Innate Immunity: A New Approach to Infection in the Post-Antibiotic Era? Surg. Infect. (Larchmt). 19, 792–803. doi: 10.1089/sur.2018.187 30277846

[B4] Batovska D. I. Todorova I. T. Tsvetkova I. V. Najdenski H. M. (2009). Antibacterial Study of the Medium Chain Fatty Acids and Their 1-Monoglycerides: Individual Effects and Synergistic Relationships. Pol. J. Microbiol. 58, 43–47. 19469285

[B5] Becam J. Walter T. Burgert A. Schlegel J. Sauer M. Seibel J.. (2017). Antibacterial Activity of Ceramide and Ceramide Analogs Against Pathogenic Neisseria. Sci. Rep. 7, 17627. doi: 10.1038/s41598-017-18071-w 29247204 PMC5732201

[B6] Bergsson G. Arnfinnsson J. Karlsson S. M. Steingrímsson O. Thormar H. (1998). *In Vitro* Inactivation of *Chlamydia Trachomatis* by Fatty Acids and Monoglycerides. Antimicrob. Agents Chemother. 42, 2290–2294. doi: 10.1128/AAC.42.9.2290 9736551 PMC105821

[B7] Bergsson G. Arnfinnsson J. Steingrímsson O. Thormar H. (2001). Killing of Gram-Positive Cocci by Fatty Acids and Monoglycerides. APMIS 109, 670–678. doi: 10.1034/j.1600-0463.2001.d01-131.x 11890570

[B8] Bibel D. J. Aly R. Shinefield H. R. (1992). Antimicrobial Activity of Sphingosines. J. Invest. Dermatol. 98, 269–273. doi: 10.1111/1523-1747.ep12497842 1545135

[B9] Biswas S. M. Chakraborty N. (2013). Shedded Artocarpus Leaves - Good Plant Sources of Natural Squalene With Potent Antioxidant and Antimicrobial Activity - Alternative to Marine Animals. J. Nat. Pharm. 4, 21–27. doi: 10.4103/2229-5119.110344

[B10] Bochkov V. N. Kadl A. Huber J. Gruber F. Binder B. R. Leitinger N. (2002). Protective Role of Phospholipid Oxidation Products in Endotoxin-Induced Tissue Damage. Nature 419, 77–81. doi: 10.1038/nature01023 12214235

[B11] Bollag W. B. Gonzales J. N. (2020). Phosphatidylglycerol and Surfactant: A Potential Treatment for COVID-19? Med. Hypotheses 144, 110277. doi: 10.1016/j.mehy.2020.110277 33254581 PMC7493731

[B12] Brasser A. J. Barwacz C. A. Dawson D. V. Brogden K. A. Drake D. R. Wertz P. W. (2011). Presence of Wax Esters and Squalene in Human Saliva. Arch. Oral. Biol. 56, 588–591. doi: 10.1016/j.archoralbio.2010.12.002 21247555 PMC3095707

[B13] Brogden N. K. Mehalick L. Fischer C. L. Wertz P. W. Brogden K. A. (2012). The Emerging Role of Peptides and Lipids as Antimicrobial Epidermal Barriers and Modulators of Local Inflammation. Skin Pharmacol. Physiol. 25, 167–181. doi: 10.1159/000337927 22538862 PMC3564229

[B14] Bron A. J. de Paiva C. S. Chauhan S. K. Bonini S. Gabison E. E. Jain S.. (2017). TFOS DEWS II Pathophysiology Report. Ocul. Surf. 15, 438–510. doi: 10.1016/j.jtos.2017.05.011 28736340

[B15] Bron A. J. Tiffany J. M. Gouveia S. M. Yokoi N. Voon L. W. (2004). Functional Aspects of the Tear Film Lipid Layer. Exp. Eye Res. 78, 347–360. doi: 10.1016/j.exer.2003.09.019 15106912

[B16] Brown S. H. Kunnen C. M. Duchoslav E. Dolla N. K. Kelso M. J. Papas E. B.. (2013). A Comparison of Patient Matched Meibum and Tear Lipidomes. Invest. Ophthalmol. Vis. Sci. 54, 7417–7424. doi: 10.1167/iovs.13-12916 24135754

[B17] Butovich I. A. (2008). On the Lipid Composition of Human Meibum and Tears: Comparative Analysis of Nonpolar Lipids. Invest. Ophthalmol. Vis. Sci. 49, 3779–3789. doi: 10.1167/iovs.08-1889 18487374 PMC2659562

[B18] Butovich I. A. (2009). The Meibomian Puzzle: Combining Pieces Together. Prog. Retin Eye Res. 28, 483–498. doi: 10.1016/j.preteyeres.2009.07.002 19660571 PMC2783885

[B19] Butovich I. A. (2013). Tear Film Lipids. Exp. Eye Res. 117, 4–27. doi: 10.1016/j.exer.2013.05.010 23769846 PMC3844095

[B20] Butovich I. A. Uchiyama E. McCulley J. P. (2007). Lipids of Human Meibum: Mass-Spectrometric Analysis and Structural Elucidation. J. Lipids Res. 48, 2220–2235. doi: 10.1194/jlr.M700237-JLR200 17626978

[B21] Butovich I. A. Wojtowicz J. C. Molai M. (2009). Human Tear Film and Meibum. Very Long Chain Wax Esters and (O-Acyl)-Omega-Hydroxy Fatty Acids of Meibum. J. Lipid Res. 50, 2471–2485. doi: 10.1194/jlr.M900252-JLR200 19535818 PMC2781319

[B22] Carson D. D. Daneo-Moore L. (1980). Effects of Fatty Acids on Lysis of *Streptococcus Faecalis* . J. Bacteriol. 141, 1122–1126. doi: 10.1128/jb.141.3.1122-1126.1980 6102557 PMC293793

[B23] Chamberlain N. R. Mehrtens B. G. Xiong Z. Kapral F. A. Boardman J. L. Rearick J. I. (1991). Correlation of Carotenoid Production, Decreased Membrane Fluidity, and Resistance to Oleic Acid Killing in *Staphylococcus Aureus* 18Z. Infect. Immun. 59, 4332–4337. doi: 10.1128/iai.59.12.4332-4337.1991 1937793 PMC259045

[B24] Chen J. (2021). Mass Spectrometric Analysis of Meibomian Gland Lipids. Methods Mol. Biol. 2306, 157–170. doi: 10.1007/978-1-0716-1410-5_11 33954946 PMC9709490

[B25] Chen J. Green-Church K. B. Nichols K. K. (2010). Shotgun Lipidomic Analysis of Human Meibomian Gland Secretions With Electrospray Ionization Tandem Mass Spectrometry. Invest. Ophthalmol. Vis. Sci. 51, 6220–6231. doi: 10.1167/iovs.10-5687 20671273 PMC3055753

[B26] Chen J. Nichols K. K. Wilson L. Barnes S. Nichols J. J. (2019). Untargeted Lipidomic Analysis of Human Tears: A New Approach for Quantification of O-Acyl-Omega Hydroxy Fatty Acids. Ocul. Surf. 17, 347–355. doi: 10.1016/j.jtos.2019.02.004 30818035 PMC7891227

[B27] Chen C. H. Wang Y. Nakatsuji T. Liu Y. T. Zouboulis C. Gallo R.. (2011). An Innate Bactericidal Oleic Acid Effective Against Skin Infection of Methicillin-Resistant *Staphylococcus Aureus*: A Therapy Concordant With Evolutionary Medicine. J. Microbiol. Biotechnol. 21, 391–399. doi: 10.4014/jmb.1011.11014 21532323

[B28] Cheung Lam A. H. Sandoval N. Wadhwa R. Gilkes J. Do T. Q. Ernst W.. (2016). Assessment of Free Fatty Acids and Cholesteryl Esters Delivered in Liposomes as Novel Class of Antibiotic. BMC Res. Notes. 9, 337. doi: 10.1186/s13104-016-2138-8 27391402 PMC4938966

[B29] Churchward C. P. Alany R. G. Snyder L. A. S. (2018). Alternative Antimicrobials: The Properties of Fatty Acids and Monoglycerides. Crit. Rev. Microbiol. 44, 561–570. doi: 10.1080/1040841X.2018.1467875 29733249

[B30] Civra A. Leoni V. Caccia C. Sottemano S. Tonetto P. Coscia A.. (2019). Antiviral Oxysterols Are Present in Human Milk at Diverse Stages of Lactation. J. Steroid Biochem. Mol. Biol. 193, 105424. doi: 10.1016/j.jsbmb.2019.105424 31302219

[B31] daSilva-Antunes K. (2013). Tear Lipids and Antibacterial Defence at the Ocular Surface. BSc (Honours) Thesis. Western Sydney University, Australia.

[B32] daSilva-Antunes K. Mudgil P. Whitehall J. (2016). "Phosphatidylcholine and Cholesteryl Esters as Antibacterial Compounds in Tears," in Australian Society for Microbiology Meeting, Perth Australia, July 3-6.

[B33] Dean A. W. Glasgow B. J. (2012). Mass Spectrometric Identification of Phospholipids in Human Tears and Tear Lipocalin. Invest. Ophthalmol. Vis. Sci. 53, 1773–1782. doi: 10.1167/iovs.11-9419 22395887 PMC3342792

[B34] Desbois A. P. Lawlor K. C. (2013). Antibacterial Activity of Long-Chain Polyunsaturated Fatty Acids Against *Propionibacterium Acnes* and *Staphylococcus Aureus* . Mar. Drugs 11, 4544–4557. doi: 10.3390/md11114544 24232668 PMC3853744

[B35] Desbois A. P. Smith V. J. (2010). Antibacterial Free Fatty Acids: Activities, Mechanisms of Action and Biotechnological Potential. Appl. Microbiol. Biotechnol. 85, 1629–1642. doi: 10.1007/s00253-009-2355-3 19956944

[B36] de Toledo-Piza A. R. de Oliveira M. I. Negri G. Mendonça R. Z. Figueiredo C. A. (2018). Polyunsaturated Fatty Acids From *Phyllocaulis Boraceiensis* Mucus Block the Replication of Influenza Virus. Arch. Microbiol. 200, 961–970. doi: 10.1007/s00203-018-1507-1 29616305

[B37] Do T. Q. Moshkani S. Castillo P. Anunta S. Pogosyan A. Cheung A.. (2008). Lipids Including Cholesteryl Linoleate and Cholesteryl Arachidonate Contribute to the Inherent Antibacterial Activity of Human Nasal Fluid. J. Immunol. 181, 4177–4187. doi: 10.4049/jimmunol.181.6.4177 18768875 PMC2597438

[B38] Dougherty J. M. McCulley J. P. (1986a). Analysis of the Free Fatty Acid Component of Meibomian Secretions in Chronic Blepharitis. Invest. Ophthalmol. Vis. Sci. 27, 52–56. 3941038

[B39] Dougherty J. M. McCulley J. P. (1986b). Bacterial Lipases and Chronic Blepharitis. Invest. Ophthalmol. Vis. Sci. 27, 486–491. 3957566

[B40] Drake D. R. Brogden K. A. Dawson D. V. Wertz P. W. (2008). Thematic Review Series: Skin Lipids. Antimicrobial Lipids at the Skin Surface. J. Lipid Res. 49, 4–11. doi: 10.1194/jlr.R700016-JLR200 17906220

[B41] Ebrahimi M. Farhadian N. Amiri A. R. Hataminia F. Soflaei S. S. Karimi M. (2022). Evaluating the Efficacy of Extracted Squalene From Seed Oil in the Form of Microemulsion for the Treatment of COVID-19: A Clinical Study. J. Med. Virol. 94, 119–130. doi: 10.1002/jmv.27273 34403141 PMC8427120

[B42] Ernandes M. J. Kagan J. C. (2021). Interferon-Independent Restriction of RNA Virus Entry and Replication by a Class of Damage-Associated Molecular Patterns. mBio 12, e00584–e00521. doi: 10.1128/mBio.00584-21 33849978 PMC8092255

[B43] Fang J. Y. Lin Y. K. Wang P. W. Alalaiwe A. Yang Y. C. Yang S. C. (2019). The Droplet-Size Effect of Squalene@Cetylpyridinium Chloride Nanoemulsions on Antimicrobial Potency Against Planktonic and Biofilm MRSA. Int. J. Nanomed. 14, 8133–8147. doi: 10.2147/IJN.S221663 PMC6790405 31632023

[B44] Fischer C. L. Blanchette D. R. Brogden K. A. Dawson D. V. Drake D. R. Hill J. R.. (2014). The Roles of Cutaneous Lipids in Host Defense. Biochim. Biophys. Acta 1841, 319–322. doi: 10.1016/j.bbalip.2013.08.012 23994607 PMC3943473

[B45] Fischer C. L. Drake D. R. Dawson D. V. Blanchette D. R. Brogden K. A. Wertz P. W. (2012). Antibacterial Activity of Sphingoid Bases and Fatty Acids Against Gram-Positive and Gram-Negative Bacteria. Antimicrob. Agents Chemother. 56, 1157–1161. doi: 10.1128/AAC.05151-11 22155833 PMC3294957

[B46] Fischer C. L. Walters K. S. Drake D. R. Dawson D. V. Blanchette D. R. Brogden K. A.. (2013). Oral Mucosal Lipids Are Antibacterial Against *Porphyromonas Gingivalis*, Induce Ultrastructural Damage, and Alter Bacterial Lipid and Protein Compositions. Int. J. Oral. Sci. 5, 130–140. doi: 10.1038/ijos.2013.28 23867843 PMC3967327

[B47] Fluhr J. W. Kao J. Jain M. Ahn S. K. Feingold K. R. Elias P. M. (2001). Generation of Free Fatty Acids From Phospholipids Regulates Stratum Corneum Acidification and Integrity. J. Invest. Dermatol. 117, 44–51. doi: 10.1046/j.0022-202x.2001.01399.x 11442748

[B48] Fox C. B. (2009). Squalene Emulsions for Parenteral Vaccine and Drug Delivery. Molecules 14, 3286–3312. doi: 10.3390/molecules14093286 19783926 PMC6254918

[B49] Frank C. L. Sitler-Elbel K. G. Hudson A. J. Ingala M. R. (2018). The Antifungal Properties of Epidermal Fatty Acid Esters: Insights From White-Nose Syndrome (WNS) in Bats. Molecules 23, 1986. doi: 10.3390/molecules23081986 PMC6222711 30096918

[B50] Fullard R. J. Tucker D. L. (1991). Changes in Human Tear Protein Levels With Progressively Increasing Stimulus. Invest. Ophthalmol. Vis. Sci. 32, 2290–2301. 2071341

[B51] Galbraith H. Miller T. B. (1973). Effect of Long Chain Fatty Acids on Bacterial Respiration and Amino Acid Uptake. J. Appl. Bacteriol. 36, 659–675. doi: 10.1111/j.1365-2672.1973.tb04151.x 4787613

[B52] Galbraith H. Miller T. B. Paton A. M. Thompson J. K. (1971). Antibacterial Activity of Long Chain Fatty Acids and the Reversal With Calcium, Magnesium, Ergocalciferol and Cholesterol. J. Appl. Bacteriol. 34, 803–813. doi: 10.1111/j.1365-2672.1971.tb01019.x 5004248

[B53] Garg S. K. Valente E. Greco E. Santucci M. B. De Spirito M. Papi M.. (2006). Lysophosphatidic Acid Enhances Antimycobacterial Activity Both *In Vitro* and *Ex Vivo* . Clin. Immunol. 121, 23–28. doi: 10.1016/j.clim.2006.06.003 16875878

[B54] Gipson I. K. Hori Y. Argueso P. (2004). Character of Ocular Surface Mucins and Their Alteration in Dry Eye Disease. Ocul. Surf. 2, 131–148. doi: 10.1016/s1542-0124(12)70149-0 17216084

[B55] González-Aldaco K. Torres-Reyes L. A. Ojeda-Granados C. José-Ábrego A. Fierro N. A. Román S. (2018). Immunometabolic Effect of Cholesterol in Hepatitis C Infection: Implications in Clinical Management and Antiviral Therapy. Ann. Hepatol. 17, 908–919. doi: 10.5604/01.3001.0012.7191 30600305

[B56] Greenway D. L. Dyke K. G. (1979). Mechanism of the Inhibitory Action of Linoleic Acid on the Growth of *Staphylococcus Aureus* . J. Gen. Microbiol. 115, 233–245. doi: 10.1099/00221287-115-1-233 93615

[B57] Hamosh M. Peterson J. A. Henderson T. R. Scallan C. D. Kiwan R. Ceriani R. L.. (1999). Protective Function of Human Milk: The Milk Fat Globule. Semin. Perinatol. 23, 242–249. doi: 10.1016/s0146-0005(99)80069-x 10405194

[B58] Harvey D. Tiffany J. Duerden J. Pandher K. Mengher L. (1987). Identification by Combined Gas Chromatography-Mass Spectrometry of Constituent Long-Chain Fatty Acids and Alcohols From the Meibomian Glands of the Rat and a Comparison With Human Meibomian Lipids. J. Chromatogr. 414, 253–263. doi: 10.1016/0378-4347(87)80051-8 3571395

[B59] Hilmarsson H. Kristmundsdóttir T. Thormar H. (2005). Virucidal Activities of Medium- and Long-Chain Fatty Alcohols, Fatty Acids and Monoglycerides Against Herpes Simplex Virus Types 1 and 2: Comparison at Different pH Levels. APMIS 113, 58–65. doi: 10.1111/j.1600-0463.2005.apm1130109.x 15676016

[B60] Hilmarsson H. Traustason B. S. Kristmundsdóttir T. Thormar H. (2007). Virucidal Activities of Medium- and Long-Chain Fatty Alcohols and Lipids Against Respiratory Syncytial Virus and Parainfluenza Virus Type 2: Comparison at Different pH Levels. Arch. Virol. 152, 2225–2236. doi: 10.1007/s00705-007-1063-5 17891329

[B61] Hoeger P. H. Schreiner V. Klaassen I. A. Enzmann C. C. Friedrichs K. (2002). Epidermal Barrier Lipids in Human Vernix Caseosa: Corresponding Ceramide Pattern in Vernix and Fetal Skin. Br. J. Dermatol. 146, 194–201. doi: 10.1046/j.1365-2133.2002.04584.x 11903227

[B62] Isaacs C. E. (2001). The Antimicrobial Function of Milk Lipids. Adv. Nutr. Res. 10, 271–285. doi: 10.1007/978-1-4615-0661-4_13 11795045

[B63] Isaacs C. E. (2005). Human Milk Inactivates Pathogens Individually, Additively, and Synergistically. J. Nutr. 135, 1286–1288. doi: 10.1093/jn/135.5.1286 15867325

[B64] Isaacs C. E. Litov R. E. Thormar H. (1995). Antimicrobial Activity of Lipids Added to Human Milk, Infant Formula, and Bovine Milk. J. Nutr. Biochem. 6, 362–366. doi: 10.1016/0955-2863(95)80003-u 12049996

[B65] Isaacs C. E. Thormar H. Pessolano T. (1986). Membrane-Disruptive Effect of Human Milk: Inactivation of Enveloped Viruses. J. Infect. Dis. 154, 966–971. doi: 10.1093/infdis/154.6.966 3491166 PMC7109787

[B66] Ji J. Sun L. Luo Z. Zhang Y. Xianzheng W. Liao Y.. (2021). Potential Therapeutic Applications of Pulmonary Surfactant Lipids in the Host Defence Against Respiratory Viral Infections. Front. Immunol. 12, 730022. doi: 10.3389/fimmu.2021.730022 PMC8503189 34646269

[B67] Kabara J. J. (1984). Antimicrobial Agents Derived From Fatty Acids. J. Ame. Oil Chem. Soc 61, 397–403. doi: 10.1007/BF02678802

[B68] Kabara J. J. Swieczkowski D. M. Conley A. J. Truant J. P. (1972). Fatty Acids and Derivatives as Antimicrobial Agents. Antimicrob. Agents Chemother. 2, 23–28. doi: 10.1128/AAC.2.1.23 4670656 PMC444260

[B69] Kalužíková A. Vrkoslav V. Harazim E. Hoskovec M. Plavka R. Buděšínský M.. (2017). Cholesteryl Esters of ω-(O-Acyl)-Hydroxy Fatty Acids in Vernix Caseosa. J. Lipid Res. 58, 1579–1590. doi: 10.1194/jlr.M075333 28576934 PMC5538280

[B70] Kamysz E. Sikorska E. Jaśkiewicz M. Bauer M. Neubauer D. Bartoszewska S.. (2020). Lipidated Analogs of the LL-37-Derived Peptide Fragment KR12-Structural Analysis, Surface-Active Properties and Antimicrobial Activity. Int. J. Mol. Sci. 21, 887. doi: 10.3390/ijms21030887 PMC7036753 32019109

[B71] Kenny J. G. Ward D. Josefsson E. Jonsson I. M. Hinds J. Rees H. H.. (2009). The *Staphylococcus Aureus* Response to Unsaturated Long Chain Free Fatty Acids: Survival Mechanisms and Virulence Implications. PLoS One 4, e4344. doi: 10.1371/journal.pone.0004344 19183815 PMC2629846

[B72] Kitahara T. Koyama N. Matsuda J. Aoyama Y. Hirakata Y. Kamihira S.. (2004). Antimicrobial Activity of Saturated Fatty Acids and Fatty Amines Against Methicillin-Resistant *Staphylococcus Aureus* . Biol. Pharm. Bull. 27, 1321–1326. doi: 10.1248/bpb.27.1321 15340213

[B73] Knapp H. R. Melly M. A. (1986). Bactericidal Effects of Polyunsaturated Fatty Acids. J. Infect. Dis. 154, 84–94. doi: 10.1093/infdis/154.1.84 3086465

[B74] Koopman J. S. Turkisk V. J. Monto A. S. Thompson F. E. Isaacson R. E. (1984). Milk Fat and Gastrointestinal Illness. Am. J. Public Health 74, 1371–1373. doi: 10.2105/ajph.74.12.1371 6095691 PMC1652700

[B75] Krogfelt K. A. Utley M. Krivan H. C. Laux D. C. Cohen P. S. (2000). Specific Phospholipids Enhance the Activity of Beta-Lactam Antibiotics Against *Pseudomonas Aeruginosa* . J. Antimicrob. Chemother. 46, 377–384. doi: 10.1093/jac/46.3.377 10980163

[B76] Lakshmaiah Narayana J. Golla R. Mishra B. Wang X. Lushnikova T. Zhang Y.. (2021). Short and Robust Anti-Infective Lipopeptides Engineered Based on the Minimal Antimicrobial Peptide KR12 of Human LL-37. ACS Infect. Dis. 7, 1795–1808. doi: 10.1021/acsinfecdis.1c00101 33890759 PMC9082781

[B77] Lam S. M. Tong L. Duan X. Petznick A. Wenk M. R. Shui G. (2014). Extensive Characterization of Human Tear Fluid Collected Using Different Techniques Unravels the Presence of Novel Lipid Amphiphiles. J. Lipid Res. 55, 289–298. doi: 10.1194/jlr.M044826 24287120 PMC3886667

[B78] Laux D. C. Corson J. M. Givskov M. Hentzer M. Møller A. Wosencroft K. A.. (2002). Lysophosphatidic Acid Inhibition of the Accumulation of *Pseudomonas Aeruginosa* PAO1 Alginate, Pyoverdin, Elastase and LasA. Microbiol. (Read.) 148 (Pt 6), 1709–1723. doi: 10.1099/00221287-148-6-1709 12055291

[B79] Lee J. T. Escobar O. H. Anouseyan R. Janisiewicz A. Eivers E. Blackwell K. E.. (2014). Assessment of Epithelial Innate Antimicrobial Factors in Sinus Tissue From Patients With and Without Chronic Rhinosinusitis. Int. Forum Allergy Rhinol. 4, 893–900. doi: 10.1002/alr.21404 25196914 PMC4289639

[B80] Lee D. Y. Huang C. M. Nakatsuji T. Thiboutot D. Kang S. A. Monestier M.. (2009). Histone H4 Is a Major Component of the Antimicrobial Action of Human Sebocytes. J. Invest. Dermatol. 129, 2489–2496. doi: 10.1038/jid.2009.106 19536143 PMC2765036

[B81] Lee J. T. Jansen M. Yilma A. N. Nguyen A. Desharnais R. Porter E. (2010). Antimicrobial Lipids: Novel Innate Defense Molecules Are Elevated in Sinus Secretions of Patients With Chronic Rhinosinusitis. Am. J. Rhinol. Allergy 24, 99–104. doi: 10.2500/ajra.2010.24.3444 20338107 PMC2925135

[B82] Lembo D. Cagno V. Civra A. Poli G. (2016). Oxysterols: An Emerging Class of Broad Spectrum Antiviral Effectors. Mol. Aspects Med. 49, 23–30. doi: 10.1016/j.mam.2016.04.003 27086126

[B83] Li Z. Yuan P. Xing M. He Z. Dong C. Cao Y.. (2013). Fatty Acid Conjugation Enhances the Activities of Antimicrobial Peptides. Recent Pat. Food Nutr. Agric. 5, 52–56. doi: 10.2174/2212798411305010008 23270392

[B84] Marquart M. E. Monds K. S. Mccormick C. C. Dixon S. N. Sanders M. E. Reed J. M.. (2007). Cholesterol as Treatment for Pneumococcal Keratitis: Cholesterol-Specific Inhibition of Pneumolysin in the Cornea. Invest. Ophthalmol. Vis. Sci. 48, 2661–2666. doi: 10.1167/iovs.07-0017 17525197 PMC2814300

[B85] Martinez J. G. Waldon M. Huang Q. Alvarez S. Oren A. Sandoval N.. (2009). Membrane-Targeted Synergistic Activity of Docosahexaenoic Acid and Lysozyme Against *Pseudomonas Aeruginosa* . Biochem. J. 419, 193–200. doi: 10.1042/BJ20081505 19105793 PMC2735766

[B86] Ma D. C. Yoon A. J. Faull K. F. Desharnais R. Zemanick E. T. Porter E. (2015). Cholesteryl Esters Are Elevated in the Lipid Fraction of Bronchoalveolar Lavage Fluid Collected From Pediatric Cystic Fibrosis Patients. PLoS One 10 (4), e0125326. doi: 10.1371/journal.pone.0125326 PMC4412572 25919295

[B87] McDermott A. M. (2013). Antimicrobial Compounds in Tears. Exp. Eye Res. 117, 53–61. doi: 10.1016/j.exer.2013.07.014 23880529 PMC3844110

[B88] Millar T. J. Mudgil P. Khanal S. (2010). “Meibomian Glands and Lipid Layer,” in The Encyclopaedia of the Eye, vol. 3 . Eds. Dartt D. A. Besharse J. Dana R R. (Oxford: Academic Press), 13–20,ISBN 9780123741981 Chapter 48.

[B89] Mishima S. Gasset A. Klyce S. D. Baum J. L. (1966). Determination of Tear Volume and Tear Flow. Invest. Ophthalmol. Vis. Sci. 5, 264–276. 5947945

[B90] Mudgil P. (2014). Antimicrobial Role of Human Meibomian Lipids at the Ocular Surface. Invest. Ophthalmol. Vis. Sci. 55, 7272–7277. doi: 10.1167/iovs.14-15512 25316725

[B91] Mudgil P. daSilva-Antunes K. Whitehall J. (2014). Oleic Acid as an Antibacterial for Treating Eye Infections. Invest. Ophthalmol. Vis. Sci. 55, 1477.

[B92] Mudgil P. Millar T. J. (2011). Surfactant Properties of Human Meibomian Lipids. Invest. Ophthalmol. Vis. Sci. 52, 1661–1670. doi: 10.1167/iovs.10-5445 21051693 10.1167/iovs.10-5445

[B93] Nakatsuji T. Kao M. C. Fang J. Y. Zouboulis C. C. Zhang L. Gallo R. L.. (2009). Antimicrobial Property of Lauric Acid Against *Propionibacterium Acnes*: Its Therapeutic Potential for Inflammatory Acne Vulgaris. J. Invest. Dermatol. 129, 2480–2488. doi: 10.1038/jid.2009.93 19387482 PMC2772209

[B94] Nakatsuji T. Kao M. C. Zhang L. Zouboulis C. C. Gallo R. L. Huang C. M. (2010). Sebum Free Fatty Acids Enhance the Innate Immune Defense of Human Sebocytes by Upregulating Beta-Defensin-2 Expression. J. Invest. Dermatol. 130, 985–994. doi: 10.1038/jid.2009.384 20032992 PMC3057125

[B95] Newburg D. S. (2005). Innate Immunity and Human Milk. J. Nutr. 135, 1308–1312. doi: 10.1093/jn/135.5.1308 15867330

[B96] Nicolaides N. Kaitaranta J. K. Rawdah T. N. Macy J. I. Boswell F. Smith R. (1981). Meibomian Gland Studies: Comparison of Steer and Human Lipids. Invest. Ophthalmol. Vis. Sci. 20, 522–536. 7194326

[B97] Nishijima K. Yoneda M. Hirai T. Takakuwa K. Enomoto T. (2019). Biology of the Vernix Caseosa: A Review. J. Obstet. Gynaecol. Res. 45, 2145–2149. doi: 10.1111/jog.14103 31507021

[B98] Numata M. Chu H. W. Dakhama A. Voelker D. R. (2010). Pulmonary Surfactant Phosphatidylglycerol Inhibits Respiratory Syncytial Virus-Induced Inflammation and Infection. Proc. Natl. Acad. Sci. U. S. A. 2010 107, 320–325. doi: 10.1073/pnas.0909361107 PMC2806703 20080799

[B99] Numata M. Kandasamy P. Nagashima Y. Fickes R. Murphy R. C. Voelker D. R. (2015). Phosphatidylinositol Inhibits Respiratory Syncytial Virus Infection. J. Lipid Res. 56, 578–587. doi: 10.1194/jlr.M055723 25561461 PMC4340305

[B100] Numata M. Kandasamy P. Nagashima Y. Posey J. Hartshorn K. Woodland D.. (2012). Phosphatidylglycerol Suppresses Influenza A Virus Infection. Am. J. Respir. Cell Mol. Biol. 46, 479–487. doi: 10.1165/rcmb.2011-0194OC 22052877 PMC3359948

[B101] Parsons J. B. Yao J. Frank M. W. Jackson P. Rock C. O. (2012). Membrane Disruption by Antimicrobial Fatty Acids Releases Low-Molecular-Weight Proteins From *Staphylococcus Aureus* . J. Bacteriol. 194, 5294–5304. doi: 10.1128/JB.00743-12 22843840 PMC3457211

[B102] Pewzner-Jung Y. Tavakoli Tabazavareh S. Grassmé H. Becker K. A. Japtokm L. Steinmann J.. (2014). Sphingoid Long Chain Bases Prevent Lung Infection by *Pseudomonas Aeruginosa* . EMBO Mol. Med. 6, 1205–1214. doi: 10.15252/emmm.201404075 25085879 PMC4197866

[B103] Pieczyński J. Szulc U. Harazna J. Szulc A. Kiewisz J. (2021). Tear Fluid Collection Methods: Review of Current Techniques. Eur. J. Ophthalmol. 31, 2245–2251. doi: 10.1177/1120672121998922 33631970

[B104] Poerio N. Bugli F. Taus F. Santucci M. B. Rodolfo C. Cecconi F.. (2017). Liposomes Loaded With Bioactive Lipids Enhance Antibacterial Innate Immunity Irrespective of Drug Resistance. Sci. Rep. 7, 45120. doi: 10.1038/srep45120 28345623 PMC5366871

[B105] Pucker A. D. Haworth K. M. (2015). The Presence and Significance of Polar Meibum and Tear Lipids. Ocul. Surf. 13, 26–42. doi: 10.1016/j.jtos.2014.06.002 25557344

[B106] Pucker A. D. Nichols J. J. (2012). Analysis of Meibum and Tear Lipids. Ocul. Surf. 10, 230–250. doi: 10.1016/j.jtos.2012.07.004 23084145

[B107] Rantamäki A. H. Seppänen-Laakso T. Oresic M. Jauhiainen M. Holopainen J. M. (2011). Human Tear Fluid Lipidome: From Composition to Function. PLoS One 6, e19553. doi: 10.1371/journal.pone.0019553 21573170 PMC3088682

[B108] Rentka A. Koroskenyi K. Harsfalvi J. Szekanecz Z. Szucs G. Szodoray P.. (2017). Evaluation of Commonly Used Tear Sampling Methods and Their Relevance in Subsequent Biochemical Analysis. Ann. Clin. Biochem. 54, 521–529. doi: 10.1177/0004563217695843 28193107

[B109] Robertson E. Burnell K. Qian F. Brogden K. A. Wertz P. Drake D. R. (2006). Synergistic Activity of Human Skin Lipids and LL37. J. Dent. Res. 85A, 2113.

[B110] Robosky L. C. Wade K. Woolson D. Baker J. D. Manning M. L. Gage D. A.. (2008). Quantitative Evaluation of Sebum Lipid Components With Nuclear Magnetic Resonance. J. Lipid Res. 49, 686–692. doi: 10.1194/jlr.D700035-JLR200 18094397

[B111] Sack R. A. Nunes I. Beaton A. Morris C. (2001). Host-Defense Mechanism of the Ocular Surfaces. Biosci. Rep. 21, 463–480. doi: 10.1023/a:1017943826684 11900322

[B112] Saulnier-Blache J.-S. (2004). L’acide Lysophosphatidique: Un Phospholipide “Bioactif “ [Lysophosphatidic Acid: A “Bioactive” Phospholipid]. Med. Sci. (Paris) 20, 799–803. doi: 10.1051/medsci/2004208-9799. French. 15361348

[B113] Schauber J. Svanholm C. Termén S. Iffland K. Menzel T. Scheppach W.. (2003). Expression of the Cathelicidin LL-37 Is Modulated by Short Chain Fatty Acids in Colonocytes: Relevance of Signalling Pathways. Gut 52, 735–741. doi: 10.1136/gut.52.5.735 12692061 PMC1773650

[B114] Sharma H. Mathew B. Nagaraj R. (2015). Engineering of a Linear Inactive Analog of Human β-Defensin 4 to Generate Peptides With Potent Antimicrobial Activity. J. Pept. Sci. 21, 501–511. doi: 10.1002/psc.2770 25810238

[B115] Sheu C. W. Freese E. (1972). Effects of Fatty Acids on Growth and Envelope Proteins of *Bacillus Subtilis.* J. Bacteriol. 111, 516–524. doi: 10.1128/jb.111.2.516-524.1972 4626502 PMC251313

[B116] Shin S. Kim H. Kang S. C. (2004). Antibacterial Activity of Various Hydroxy Fatty Acids Bioconveted by *Pseudomonas Aeruginosa* PR3. J. Appl. Biol. Chem. 47, 205–208.

[B117] Silva R. C. D. Colleran H. L. Ibrahim S. A. (2021). Milk Fat Globule Membrane in Infant Nutrition: A Dairy Industry Perspective. J. Dairy Res. 88, 105–116. doi: 10.1017/S0022029921000224 33722311

[B118] Sirigu P. Shen R. L. Pinto da Silva P. (1992). Human Meibomian Glands: The Ultrastructure of Acinar Cells as Viewed by Thin Section and Freeze-Fracture Transmission Electron Microscopies. Invest. Ophthalmol. Vis. Sci. 33, 2284–2292. 1607240

[B119] Smith K. R. Thiboutot D. M. (2008). Thematic Review Series: Skin Lipids. Sebaceous Gland Lipids: Friend or Foe? J. Lipid Res. 49, 271–281. doi: 10.1194/jlr.R700015-JLR200 17975220

[B120] Speert D. P. Wannamaker L. W. Gray E. D. Clawson C. C. (1979). Bactericidal Effect of Oleic Acid on Group A Streptococci: Mechanism of Action. Infect. Immun. 26, 1202–1210. doi: 10.1128/iai.26.3.1202-1210.1979 393631 PMC414747

[B121] Sprong R. C. Hulstein M. F. van der Meer R. (2001). Bactericidal Activities of Milk Lipids. Antimicrob. Agents Chemother. 45, 1298–1301. doi: 10.1128/AAC.45.4.1298-1301.2001 11257052 PMC90461

[B122] Subramanian C. Frank M. W. Batte J. L. Whaley S. G. Rock C. O. (2019). Oleate Hydratase From *Staphylococcus Aureus* Protects Against Palmitoleic Acid, the Major Antimicrobial Fatty Acid Produced by Mammalian Skin. tJ. Biol. Chem. 294, 9285–9294. doi: 10.1074/jbc.RA119.008439 PMC6556589 31018965

[B123] Takigawa H. Nakagawa H. Kuzukawa M. Mori H. Imokawa G. (2005). Deficient Production of Hexadecenoic Acid in the Skin Is Associated in Part With the Vulnerability of Atopic Dermatitis Patients to Colonization by *Staphylococcus Aureus* . Dermatology 211, 240–248. doi: 10.1159/000087018 16205069

[B124] Tavakoli Tabazavareh S. Seitz A. Jernigan P. Sehl C. Keitsch S. Lang S.. (2016). Lack of Sphingosine Causes Susceptibility to Pulmonary *Staphylococcus Aureus* Infections in Cystic Fibrosis. Cell Physiol. Biochem. 38, 2094–2102. doi: 10.1159/000445567 27184795

[B125] Thormar H. Isaacs C. E. Brown H. R. Barshatzky M. R. Pessolano T. (1987). Inactivation of Enveloped Viruses and Killing of Cells by Fatty Acids and Monoglycerides. Antimicrob. Agents Chemother. 31, 27–31. doi: 10.1128/AAC.31.1.27 3032090 PMC174645

[B126] Tiffany J. M. (1987). The Lipid Secretion of the Meibomian Glands. Adv. Lipid Res. 22, 1–62. doi: 10.1016/b978-0-12-024922-0.50005-9 3328487

[B127] Tiffany J. M. (2008). The Normal Tear Film. Dev. Ophthalmol. 41, 1–20. doi: 10.1159/000131066 18453758

[B128] Tollin M. Bergsson G. Kai-Larsen Y. Lengqvist J. Sjövall J. Griffiths W.. (2005). Vernix Caseosa as a Multi-Component Defence System Based on Polypeptides, Lipids and Their Interactions. Cell. Mol. Life Sci. 62, 2390–2399. doi: 10.1007/s00018-005-5260-7 16179970 PMC2315785

[B129] Tricarico P. M. Caracciolo I. Gratton R. D’Agaro P. Crovella S. (2019). 25-Hydroxycholesterol Reduces Inflammation, Viral Load and Cell Death in ZIKV-Infected U-87 MG Glial Cell Line. Inflammopharmacology 27, 621–625. doi: 10.1007/s10787-018-0517-6 30019309

[B130] van Smeden J. Boiten W. A. Hankemeier T. Rissmann R. Bouwstra J. A. Vreeken R. J. (2014). Combined LC/MS-Platform for Analysis of All Major Stratum Corneum Lipids, and the Profiling of Skin Substitutes. Biochim. Biophys. Acta 1841, 70–79. doi: 10.1016/j.bbalip.2013.10.002 24120918

[B131] Wertz P. W. (2021). Roles of Lipids in the Permeability Barriers of Skin and Oral Mucosa. Int. J. Mol. Sci. 22, v5229. doi: 10.3390/ijms22105229 PMC8155912 34063352

[B132] Wertz P. W. de Szalay S. (2020). Innate Antimicrobial Defense of Skin and Oral Mucosa. Antibiot. (Basel) 9, 159. doi: 10.3390/antibiotics9040159 PMC7235825 32260154

[B133] Willcox M. D. P. Argüeso P. Georgiev G. A. Holopainen J. M. Laurie G. W. Millar T. J.. (2017). TFOS DEWS II Tear Film Report. Ocul. Surf. 15, 366–403. doi: 10.1016/j.jtos.2017.03.006 28736338 PMC6035753

[B134] Wille J. J. Kydonieus A. (2003). Palmitoleic Acid Isomer (C16:1delta6) in Human Skin Sebum Is Effective Against Gram-Positive Bacteria. Skin Pharmacol. Appl. Skin Physiol. 16, 176–187. doi: 10.1159/000069757 12677098

[B135] Won S. R. Hong M. J. Kim Y. M. Li C. Y. Kim J. W. Rhee H. I. (2007). Oleic Acid: An Efficient Inhibitor of Glucosyltransferase. FEBS Lett. 581, 4999–5002. doi: 10.1016/j.febslet.2007.09.045 17910959

[B136] Wood P. L. (2020). Fatty Acyl Esters of Hydroxy Fatty Acid (FAHFA) Lipid Families. Metabolites 10, 512. doi: 10.3390/metabo10120512 PMC7766670 33348554

[B137] Wood P. L. Donohue M. N. Cebak J. E. Beckmann T. G. Treece M. Johnson J. W.. (2018). Tear Film Amphiphilic and Anti-Inflammatory Lipids in Bovine Pink Eye. Metabolites 8, 81. doi: 10.3390/metabo8040081 PMC6316582 30469369

[B138] Yang D. Pornpattananangkul D. Nakatsuji T. Chan M. Carson D. Huang C. M.. (2009). The Antimicrobial Activity of Liposomal Lauric Acids Against *Propionibacterium Acnes* . Biomaterials 30, 6035–6040. doi: 10.1016/j.biomaterials.2009.07.033 19665786 PMC2735618

[B139] Yoon B. K. Jackman J. A. Valle-González E. R. Cho N. J. (2018). Antibacterial Free Fatty Acids and Monoglycerides: Biological Activities, Experimental Testing, and Therapeutic Applications. Int. J. Mol. Sci. 19, 1114. doi: 10.3390/ijms19041114 PMC5979495 29642500

[B140] Zheng C. J. Yoo J. S. Lee T. G. Cho H. Y. Kim Y. H. Kim W. G. (2005). Fatty Acid Synthesis Is a Target for Antibacterial Activity of Unsaturated Fatty Acids. FEBS Lett. 579, 5157–5162. doi: 10.1016/j.febslet.2005.08.028 16146629

[B141] Zhou Q. D. Chi X. Lee M. S. Hsieh W. Y. Mkrtchyan J. J. Feng A. C.. (2020). Interferon-Mediated Reprogramming of Membrane Cholesterol to Evade Bacterial Toxins. Nat. Immunol. 21, 746–755. doi: 10.1038/s41590-020-0695-4 32514064 PMC7778040

